# Exact solutions of the (3+1)-generalized fractional nonlinear wave equation with gas bubbles

**DOI:** 10.1038/s41598-024-52249-3

**Published:** 2024-01-22

**Authors:** Aly R. Seadawy, Asghar Ali, Ali Altalbe, Ahmet Bekir

**Affiliations:** 1 Mathematics Department, Faculty of Science, Taibah University, Al-Madinah Al-Munawarah, 41411 Kingdom of Saudi Arabia; 2 Department of Mathematics, University of Education, Multan Campus, Lahore, Pakistan; 3 Department of Computer Science, Prince Sattam Bin Abdulaziz University, Al-Kharj, 11942 Saudi Arabia; 4 Faculty of Computing and Information Technology, King Abdulaziz University, Jeddah, 21589 Saudi Arabia; 5 Neighbourhood of Akcaglan, Imarli Street, Number:28/4, 26030 Eskisehir, Turkey

**Keywords:** Applied mathematics, Computational science

## Abstract

In this manuscript, we implement the travelling wave solutions of the fractional (3+1) generalized computational nonlinear wave equation with gas bubbles via application of five mathematical methods. Liquids with gas bubbles primarily arise in various applications like science, engineering, and mathematical physics. The obtained solitary waves solutions have fruitful applications in engineering, science, life, nature and physics. Several novel soliton solutions of concerned model are established in the form of hyperbolic, trigonometric, exponential and rational functions. To handle all calculations and verification of obtained results, computational software Mathematica 12.1 is used. For the demonstration of the physical behaviour of concern model, some solutions are plotted graphical in 2-dimensional and 3-dimensional by imparting specific values to the parameters under constrain conditions. Finally, we intrigue both two and three dimensional to explain the physical behavior of the model.

## Introduction

The exploration of nonlinear computational partial differential equation solutions is perilous in understanding numerous physical situations in several scientific and engineering applications. As a result, numerous logical and numerical methods have been used to tackle a diversity of such problems, including the generalized Kudryashov^1^, sine-cosine^2^, sine-Gordon expansion, extended auxiliary equation^3,4^, direct algebra^5^ , Safdar sub-equation^6^, generalized Riccati method^7^ and many more^8–26^. These approaches intensely depend on wave transformation techniques. However, other analytical methods didn’t depend on the wave transforms approach, invariant subspace method^12,13^, Lie symmetry method^8,11^, reduction method^14,15^, etc.^32–41^. The study of bubbling liquids and their applications in numerous disciplines of engineering and medical sciences has annoyed the curiosity of several scholars for periods. Most bubbles with uniform radius are elucidated by a fourth-order linear partial differential equation for convinced physical phenomena in isothermal bubbly liquids^27–31,42–49^.

Consider the liquid with the gas bubbles model as^27,28^;

1 $$\begin{aligned} \left( Q_{t}+\delta _1 QQ_{x}+\delta _2Q_{xxx}+\delta _{3}Q_{x}\right) _{x}+\delta _4Q_{yy}+\delta _5Q_{zz}=0, \end{aligned}$$

where $Q_{t}$ and $QQ_{x}$ is used to perform a role in the evolution of time and the steepening of the wave and Q is wave amplitude. The parameters $\delta _1,\delta _2,\delta _3,\delta _4$ and $\delta _5$ are represented the bubble-liquid-nonlinearity, the bubble liquid-viscosity The y transverse perturbation and the z transverse perturbation.The scrutiny of a generalized (3+1)-dimensional nonlinear wave equation that simulates a variety of nonlinear processes that transpire in liquids with gas bubbles will be accomplished.

Furthermore, almost studies on the logical and numerical blend (solution) of generalized nonlinear model Eq. (1) with gas bubbles have been explored in the literature, for example, the bilinear formalism and soliton solutions using Hirota bilinear method^21^, Assemble mixed rogue wave-stripe solitons and mixed lump-stripe solitons^23^, the binary Bell polynomials obtaining the bilinear form of this model^25^, and the solitons and lumps solution for the generalized nonlinear wave^26^. There are numerous fractional derivative operators in fractional calculus, such as the Caputo derivative, Grunwald derivative, Riemann–Liouville derivative, and so on.

Therefore, the chief focus of this research is to accomplish waves solutions of Eq. (1) via five mathematical methods^50–54^, these methods are called ESE method^50^, modified extended AEM approach^51^, $(G'/G)$-expansion scheme^52^, $Exp(-\Psi (\phi ))$-expansion method^53^ and modified F-expansion method^54^ respectively. The investigated our solutions are in different types like exponential, trigonometric, hyperbolic and rational forms and are totally d new solutions as compared to exist in previous literature by using the different techniques of distinct authors on this model^21,23,25,26^.

The remaining arrangement of research work as: In “Description of accordant fractional derivatives” some definition of common fractional derivatives, properties of the accordant derivative. In “Description of mathematical methods”, proposed mathematical methods are described. In “Applications”, soliton solutions of Eq. (1) are established by application of five mathematical method. In “Discussion of the results”, Discussion of results has been been mentioned. In “Conclusion”, conclusion of the work has been discussed.

## Description of accordant fractional derivatives

This segment will start by defining the bulk common partial (fractional) derivative definitions, like: the Riemann–Liouville, Caputo, and Grunwald–Letnikov definitions^42^.

### Definition 1

(*Riemann Liouville*)

2 $$\begin{aligned} L_x^{\mu }Q(x)=\frac{\left( \frac{d}{\text {dx}}\right) ^m \int _0^x Q z (x-z)^{-\mu +m-1} \, dz}{\Gamma (m-\mu )},~~m-1<\mu \le m \end{aligned}$$

### Definition 2

(*Caputo* )

3 $$\begin{aligned} L_x^{\mu }Q(x)=\frac{\int _0^x Q^m(z) (x-z)^{-\mu +m-1} \, dz}{\Gamma (m-\mu )},~~~m-1<\mu \le m \end{aligned}$$

### Definition 3

(*Grunwald–Letnikov*)

4 $$\begin{aligned} L_{\text {a x}}^{\mu }Q(x)= \underset{\gamma \rightarrow 0}{\text {Limit}} \gamma ^{-\mu }\sum _{i\rightarrow 0}^{\frac{x-a}{\gamma }} (-1)^i Q (x-\text {i}\gamma ) \left( \begin{array}{c} \mu \\ i \\ \end{array} \right) \end{aligned}$$

### Definition 4

(*Accordant divided (fractional) derivative* ))

Let a function $Q:[0,\infty ]\rightarrow ()$ then the Accordant divided (fractional) derivative of Q order $\delta$ is as follow:

5 $$\begin{aligned} L_z^{\mu }Q(z)=\underset{\tau \rightarrow 0}{\text {Limit}}\left( \frac{Q \left( \tau \text {z}^{1-\mu }+z\right) -Q (z)}{\tau }\right) ,~~~z>0,~\mu \epsilon (0,1) \end{aligned}$$

### Theorem 2.1

(^42^)

Let R(z),Q(z) are $\mu$-difference at a point $z>0$ and $\mu \epsilon (0,1)$. Then

1:   $$L_z^{\mu } \left( \alpha _2 Q z+\alpha _1 R z\right) =\alpha _2 Q z L_z^{\mu }+\alpha _1 R z L_z^{\mu }$$

2:    $$z^h L_z^{\mu }=h \overset{h-\mu }{z}$$

3:   $L_z^{\mu }(\lambda )=0$     for all constant function $Q(z)=\lambda$

4:   $$L_z^{\mu }\left( R(z) (Q (z)\right)$$=R(z) $$L_z^{\mu }\left( (Q (z)\right)$$+Q(z) $$L_z^{\mu }\left( (R (z)\right)$$

5: If Q(z) is differentile then $L_z^{\mu }(R)z=\frac{(\text {dR} (z)) z^{1-\mu }}{\text {dz}}$

## Description of mathematical methods

Consider the general nonlinear FDEs

6 $$\begin{aligned} F_1\left( U,U_{x},U_{t},D_{t}^{\alpha _1},... \right) =0,~~~~0<\alpha _1\le 1 \end{aligned}$$

Let the fractional transformation,

7 $$\begin{aligned} U= U(\xi ), ~~~~~~ \xi =\left( \frac{x^{\alpha _1}}{\alpha _1}-\omega \frac{t^{\alpha _1}}{\alpha _1}\right) \end{aligned}$$

Put Eq. (7) into Eq. (6),

8 $$\begin{aligned} F_2\left( U, U', U'', U''',... \right) =0, \end{aligned}$$

### Extended simple equation method

Let (8) has solutiion,

9 $$\begin{aligned} U(\xi )= \sum ^N_ {i=-N}A_i\varPsi ^i(\xi ) \end{aligned}$$

Let $$\varPsi$$ satisfy,

10 $$\begin{aligned} \varPsi '= c_0+c_1\varPsi + c_2\varPsi ^2+c_3\varPsi ^3 \end{aligned}$$

The general solutions of new simple ansatz Eq. (10) are as following

11 $$\begin{aligned} \varPsi (\xi )= -\left( \frac{c_1-\sqrt{4 c_0 c_2-c_1^2} \tan \left( \frac{1}{2} \sqrt{4 c_0 c_2-c_1^2} \left( \xi +\xi _0\right) \right) }{2 c_2}\right) ,~~4c_0c_2>c_1^2,~~~c_3=0 \end{aligned}$$

If $c_0=0,~c_3=0$ , then simple ansatz Eq. (10) reduces to Bernoulli equation, which has the following solutions:

12 $$\begin{aligned} \varPsi (\xi )= \frac{c_1 \exp \left( c_1 \left( \xi +\xi _0\right) \right) }{1-c_2 \exp \left( c_1 \left( \xi +\xi _0\right) \right) },~c_1>0 \end{aligned}$$

13 $$\begin{aligned} \varPsi (\xi )= -\frac{c_1 \exp \left( c_1 \left( \xi +\xi _0\right) \right) }{c_2 \exp \left( c_1 \left( \xi +\xi _0\right) \right) +1},~c_1<0 \end{aligned}$$

If $c_1=0,~c_3=0$, then the ansatz Eq. (10) reduces to Riccati equation, which has the following solutions:

14 $$\begin{aligned} \varPsi (\xi )=\frac{\sqrt{c_0 c_2} \tan \left( \sqrt{c_0 c_2} \left( \xi +\xi _0\right) \right) }{c_2},~~c_0c_2>0 \end{aligned}$$

15 $$\begin{aligned} \varPsi (\xi )=\frac{\sqrt{-c_0 c_2} \tanh \left( \sqrt{-c_0 c_2} \left( \xi +\xi _0\right) \right) }{c_2},~~c_0c_2<0 \end{aligned}$$

Substitute Eq. (9) along with Eq. (10) into Eq. (8), obtained a system of equations which can be solved to achieve the required solution of Eq. (8) with help of Eqs. (11–15).

### Modified extended auxiliary equation mapping method

Let solution of Eq. (8) is

16 $$\begin{aligned} U= \sum ^N_ {i=0}A_i \varPsi ^i+\sum ^{-N}_ {i=-1}B_{-i} \varPsi ^i+ \sum ^N_ {i=2}C_i \varPsi ^{i-2} \varPsi ^{'}+ \sum ^N_ {i=1}D_i\left( \frac{ \varPsi ^{'}}{ \varPsi }\right) ^i \end{aligned}$$

Let $$\Psi '$$ satisfiy,

17 $$\begin{aligned} \varPsi ' = \sqrt{\beta _1\varPsi ^2+\beta _2\varPsi ^3+\beta _3\varPsi ^4} \end{aligned}$$

The ansatz Eq. (17) has the following solutions as;

18 $$\begin{aligned} \varPsi&=-\left( \frac{\beta _1 \left( \epsilon \coth \left( \frac{1}{2} \sqrt{\beta _1} \left( \xi +\xi _0\right) \right) +1\right) }{\beta _2}\right) ,~\beta _1>0,~\epsilon =1~ or-1,~\beta _2^2-4 \beta _1 \beta _3=0 \end{aligned}$$

19 $$\begin{aligned} \varPsi&=-\sqrt{\frac{\beta _1}{4 \beta _3}} \left( \frac{\epsilon \sinh \left( \sqrt{\beta _1} \left( \xi +\xi _0\right) \right) }{\cosh \left( \sqrt{\beta _1} \left( \xi +\xi _0\right) \right) +\eta }+1\right) ,~\beta _1>0,~\beta _3>0,~\beta _2=\sqrt{4 \beta _1 \beta _3}\nonumber \\(\epsilon ,\eta )&=(1,1),(1,-1),~(-1,1),~(-1,-1) \end{aligned}$$

20 $$\begin{aligned} \varPsi&=-\left( \frac{\beta _1 \left( \frac{\epsilon \left( \sinh \left( \sqrt{\beta _1} \left( \xi +\xi _0\right) \right) +p\right) }{\cosh \left( \sqrt{\beta _1} \left( \xi +\xi _0\right) \right) +\eta \sqrt{p^2+1}}+1\right) }{\beta _2}\right) ,~\beta _1>0,\nonumber \\(\epsilon ,\eta )&=(1,1),~(1,-1),~(-1,1),~(-1,-1) \end{aligned}$$

Substitute Eq. (16) along with Eq. (17) into Eq. (8), obtained a system of equations which can be solved to achieve the required solutionof Eq. (8) with help of Eqs. (18–20).

### The $(G'/G)$-expansion method

Let Eq. (8) has solutiion,

21 $$\begin{aligned} U=A_{0}+\sum _{i=1}^N A_{i}\left( \frac{G'}{G}\right) \end{aligned}$$

L Let $G(\xi )$ obeys the second order ODE as

22 $$\begin{aligned} G''+\lambda _1 G'+\mu _1 G=0, \end{aligned}$$

Put Eq. (21) with Eq. (22) in Eq. (8), obtained a system of equations having the following solutions cases.

**CASE I**: When  $$\lambda _1^2-4 \mu _1 >0$$

23 $$\begin{aligned} \left( G'/G\right) =\frac{\sqrt{\lambda _1^2-4 \mu _1} \left( \xi P_1 \sinh \left( \frac{1}{2} \sqrt{\lambda _1^2-4 \mu _1}\right) +\xi P_2 \cosh \left( \frac{1}{2} \sqrt{\lambda _1^2-4 \mu _1}\right) \right) }{2 \left( \xi P_2 \sinh \left( \frac{1}{2} \sqrt{\lambda _1^2-4 \mu _1}\right) +\xi P_1 \cosh \left( \frac{1}{2} \sqrt{\lambda _1^2-4 \mu _1}\right) \right) }-\frac{\lambda _1}{2} \end{aligned}$$

**CASE II**: When  $\lambda _1^2-4 \mu _1 <0$

24 $$\begin{aligned} \left( G'/G\right) =\frac{\sqrt{4 \mu _1-\lambda _1^2} \left( \xi P_2 \cos \left( \frac{1}{2} \sqrt{4 \mu _1-\lambda _1^2}\right) -\xi P_1 \sin \left( \frac{1}{2} \sqrt{4 \mu _1-\lambda _1^2}\right) \right) }{2 \left( \xi P_2 \sin \left( \frac{1}{2} \sqrt{4 \mu _1-\lambda _1^2}\right) +\xi P_1 \cos \left( \frac{1}{2} \sqrt{4 \mu _1-\lambda _1^2}\right) \right) }-\frac{\lambda _1}{2} \end{aligned}$$

**CASE III**: When  $\lambda _1^2-4 \mu _1 =0$

25 $$\begin{aligned} \left( G'/G\right) =\left( \frac{P_2}{\xi \text {P}_2+P_1}-\frac{\lambda _1}{2}\right) \end{aligned}$$

By substituting all solutions of Eq. (22) into Eq. (23), we obtained the required solutions of Eq. (8)

### The $Exp(-\varPsi (\xi ))$-expansion method

Let solution of Eq. (8) is

26 $$\begin{aligned} U=A_{N}\left( Exp(-\varPsi (\xi \right) ^N+..., A_{N} \ne 0 \end{aligned}$$

Let

27 $$\begin{aligned} \varPsi '=Exp(-\varPsi (\xi ))+\mu _1 Exp(\varPsi (\xi ))+\lambda _1 \end{aligned}$$

When  $\lambda _1^2-4 \mu _1 >0$,   $\mu _1\ne 0$ then Eq. (27) has the following solution

28 $$\begin{aligned} \varPsi =\log \left( \frac{-\sqrt{\lambda _1^2-4 \mu _1} \tanh \left( \frac{1}{2} \sqrt{\lambda _1^2-4 \mu _1} (\xi +\chi )\right) -\lambda _1}{2 \mu _1}\right) \end{aligned}$$

When  $\lambda _1^2-4 \mu _1 >0$,   $\mu _1=0$, then Eq. (27) has the following solution

29 $$\begin{aligned} \varPsi =-\log \left( \frac{\lambda _1 }{e^{\lambda _1 \left( \xi +\chi \right) }-1}\right) \end{aligned}$$

When  $\lambda _1^2-4 \mu _1 =0$,   $\lambda _1\ne 0$, then Eq. (27) has the following solution

30 $$\begin{aligned} \varPsi =\log \left( \frac{2-2 \lambda _1 \left( \xi +\chi \right) }{\lambda _1^2 \left( \xi +\chi \right) }\right) \end{aligned}$$

When  $\lambda _1^2-4 \mu _1 =0$,   $\mu _1, \lambda _1=0$, then Eq. (27) has the following solution

31 $$\begin{aligned} \varPsi =\log (\xi +\chi ) \end{aligned}$$

When  $\lambda _1^2-4 \mu _1 <0$, then Eq. (27) has the following solution

32 $$\begin{aligned} \varPsi =\left( \log \left( -\frac{\sqrt{4 \mu _1-\lambda _1^2} \tan \left( \frac{1}{2} \sqrt{4 \mu _1-\lambda _1^2} (\xi +\chi )\right) +\lambda _1}{2 \mu _1}\right) \right) \end{aligned}$$

By substituting all solutions of Eq. (27) into Eq. (26), we obtained the required solutions of Eq. (8)

### Modified F-expansion method

 Let solution of (8) is:

33 $$\begin{aligned} U= a_{0}+\sum ^N_ {i=1}a_iF^i(\xi )+\sum ^N_ {i=1}b_iF^{-i}(\xi ) \end{aligned}$$

Let *F* obliges,

34 $$\begin{aligned} F' = A+BF+ CF^2. \end{aligned}$$

The relation between *A*, *B*, *C* corresponding values of $F(\xi )$ in Eq. (34) is given as;

**Table:** 

Values of *A*, *B*, *C*	$F(\xi )$
$A=0$, $B=1$, $C=-1$	$\frac{1}{2}+\frac{1}{2}\tanh (\frac{1}{2}\xi )$
$A=0$, $B=-1$, $C=1$	$\frac{1}{2}-\frac{1}{2}\coth (\frac{1}{2}\xi )$
$A=\frac{1}{2}$, $B=0$, $C=-\frac{1}{2}$	$\coth (\xi )\pm \csc h(\xi )$
$A=1$, $B=0$, $C=-1$	$\tanh (\xi ),\coth (\xi )$
$A=\frac{1}{2}$, $B=0$, $C=\frac{1}{2}$	$\sec (\xi )+\tan (\xi )$
$A=-\frac{1}{2}$, $B=0$, $C=-\frac{1}{2}$	$\sec (\xi )-\tan (\xi )$
$A=1(-1)$, $B=0$, $C=1(-1)$	$\tan (\xi )(\cot (\xi ))$
$A=0$, $B=0$, $C\ne 0$	$-\frac{1}{C\xi +\epsilon }, (\epsilon$ is arbitary constant)
$A\ne 0$, $B=0$, $C=0$	$A\xi$
$A\ne 0$, $B\ne 0$, $C=0$	$\frac{\exp (B\xi )-A}{B}$

By substituting all solutions of Eq. (34) into Eq. (33), we obtained the required solutions of Eq. (8)

## Applications

This segment will present several type results for the fractional (3+1)-dimensional (aspect) generalized (computational) wave model of liquids with gas bubbles Eq. (1) via applications of five mathematical methods. Now, utilizing the definition of the accordant fractional derivative in Eq. (1) to achieve

35 $$\begin{aligned} L_x^{\mu } \left( \delta _1 L_x^{\mu }Q^2+ L_t^{\mu }Q+\delta _2 L_x^{3 \mu } Q+\delta _3 L_x^{\mu }\right) Q+\delta _4 L_{\text {yy}}^{2 \mu }Q+\delta _5 L_{\text {zz}}^{2 \mu }Q=0 \end{aligned}$$

Consider wave transformation,

36 $$\begin{aligned} Q(x,y,z,t)=U,~~\xi =k\left( \alpha \frac{ x^{\mu }}{\mu }+\beta \frac{ y^{\mu }}{\mu }+\gamma \frac{ z^{\mu }}{\mu }-\omega \frac{ t^{\mu }}{\mu }\right) \end{aligned}$$

Subsitute Eq. (36) in Eq. (35),

37 $$\begin{aligned} \alpha ^4 \delta _3 k^2 U''+\alpha ^2 \delta _1 U^2+U \left( \alpha ^2 \delta _3-\alpha \omega +\beta ^2 \delta _4+\gamma ^2 \delta _5\right) =0 \end{aligned}$$

### Application of extended simple equation method

Let Eq. (37) has solution as;

38 $$\begin{aligned} U= A_2 \varPsi ^2 +A_1 \varPsi +\frac{A_{-1}}{\varPsi }+ \frac{A_{-2}}{\varPsi ^2 }+A_0 \end{aligned}$$

Putting Eq. (38) with (10) into (37),

**CASE 1**: $$c_3=0$$,

**FAMILY-I**

39 $$\begin{aligned}&A_0=-\frac{\alpha ^2 \left( c_1^2+2 c_0 c_2\right) \delta _3 k^2}{\delta _1},~A_{-1}=0,~A_1=-\frac{6 \alpha ^2 c_1 c_2 \delta _3 k^2}{\delta _1},~A_{-2}=0,\nonumber \\ {}&~A_2=-\frac{6 \alpha ^2 c_2^2 \delta _3 k^2}{\delta _1},~\omega =\frac{\alpha ^2 \delta _3+\beta ^2 \delta _4+\gamma ^2 \delta _5+\alpha ^4 c_1^2 \delta _3 \left( -k^2\right) +4 \alpha ^4 c_0 c_2 \delta _3 k^2}{\alpha } \end{aligned}$$

Put (39) in (38),

40 $$\begin{aligned} U_{1}=\left( \frac{\alpha ^2 \left( c_1^2-4 c_0 c_2\right) \delta _3 k^2 \left( 3 \sec ^2\left( \frac{1}{2} \sqrt{4 c_0 c_2-c_1^2} (\xi +\chi )\right) -2\right) }{2 \delta _1}\right) ,~4c_0c_2>c_1^{2}. \end{aligned}$$

**FAMILY-II**

41 $$\begin{aligned}&A_0=-\frac{\alpha ^2 \left( c_1^2+2 c_0 c_2\right) \delta _3 k^2}{\delta _1},~A_{-1}=-\frac{6 \alpha ^2 c_0 c_1 \delta _3 k^2}{\delta _1},~A_1=0,~A_{-2}=-\frac{6 \alpha ^2 c_0^2 \delta _3 k^2}{\delta _1},~A_2=0,\nonumber \\ {}&\omega =\frac{\alpha ^2 \delta _3+\beta ^2 \delta _4+\gamma ^2 \delta _5+\alpha ^4 c_1^2 \delta _3 \left( -k^2\right) +4 \alpha ^4 c_0 c_2 \delta _3 k^2}{\alpha } \end{aligned}$$

**Figure 1:**
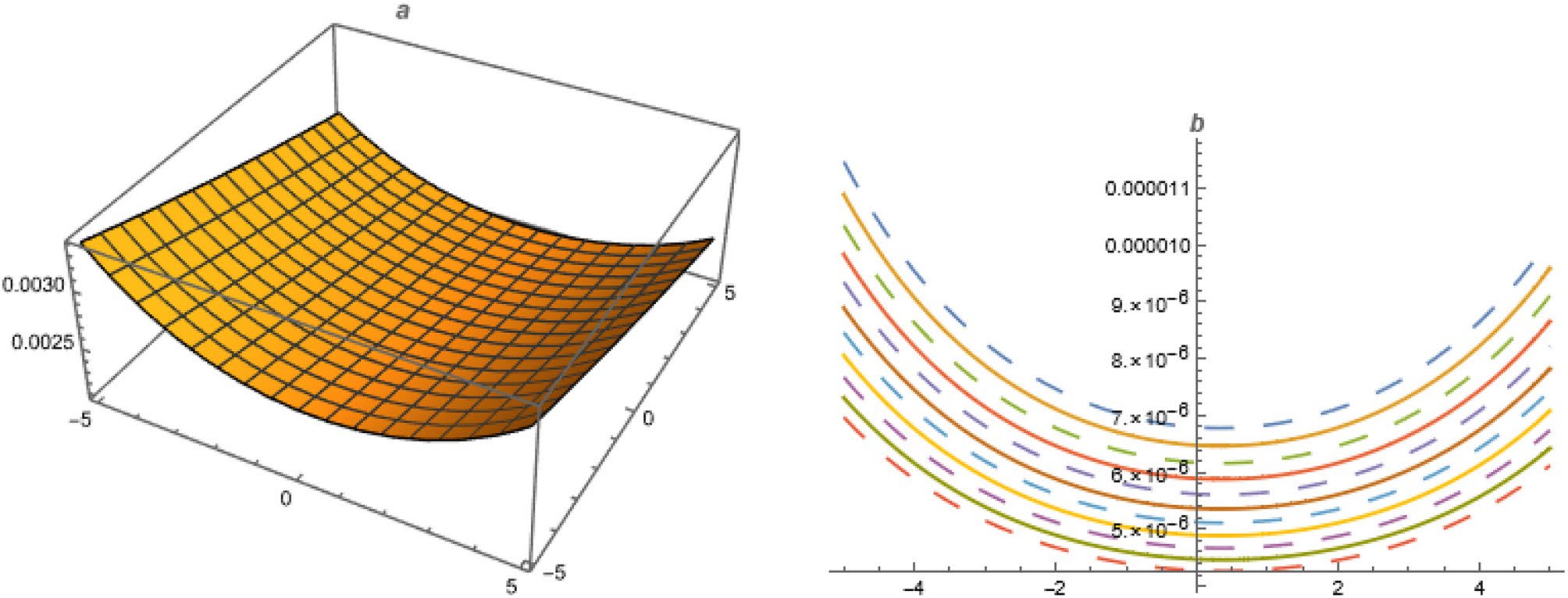
Solution $$U_{1}$$ with $$\alpha =1,~\beta =0.3,~c_0=1,~c_1=1,~c_2=1,~\gamma =-1,~\delta _1=0.03,~\delta _2=1,~\delta _3=-1,~\delta _4=-0.02,~\delta _5=1.01,~k=0.003,~\mu =1,~\chi =1,~y=1,~z=1$$.

Substitute (41) in (38),

42 $$\begin{aligned} U_{2}&=\left( \frac{\alpha ^2 \delta _3 k^2 \left( \frac{12 c_0 c_2 c_1}{c_1-\sqrt{4 c_0 c_2-c_1^2} \tan \left( \frac{1}{2} \sqrt{4 c_0 c_2-c_1^2} (\xi +\chi )\right) }+2 c_0 c_2 \left( -\frac{12 c_0 c_2}{\left( c_1-\sqrt{4 c_0 c_2-c_1^2} \tan \left( \frac{1}{2} \sqrt{4 c_0 c_2-c_1^2} (\xi +\chi )\right) \right) {}^2}-1\right) -c_1^2\right) }{\delta _1}\right) ,\nonumber \\ {}&\quad ~4c_0c_2>c_1^{2} \end{aligned}$$

**CASE 2:**   $$c_0=c_3=0$$,

43 $$\begin{aligned}&A_0=-\frac{\alpha ^2 c_1^2 \delta _3 k^2}{\delta _1},~A_{-2}=0,~A_{-1}=0,~A_2=-\frac{6 \alpha ^2 c_2^2 \delta _3 k^2}{\delta _1},\nonumber \\ {}&~A_1=-\frac{6 \alpha ^2 c_1 c_2 \delta _3 k^2}{\delta _1},~\omega =\frac{\alpha ^2 \delta _3+\beta ^2 \delta _4+\gamma ^2 \delta _5+\alpha ^4 c_1^2 \delta _3 \left( -k^2\right) }{\alpha } \end{aligned}$$

Put (43) in (38),

44 $$\begin{aligned}{} & {} U_{3} =\left( -\frac{\alpha ^2 c_1^2 \delta _3 k^2 \left( c_2 e^{c_1 (\xi +\chi )} \left( c_2 e^{c_1 (\xi +\chi )}+4\right) +1\right) }{\delta _1 \left( c_2 e^{c_1 (\xi +\chi )}-1\right) ^2}\right) ,~c_1>0. \end{aligned}$$

45 $$\begin{aligned}{} & {} U_{4} =\left( -\frac{\alpha ^2 c_1^2 \delta _3 k^2 \left( c_2 e^{c_1 (\xi +\chi )} \left( c_2 e^{c_1 (\xi +\chi )}-4\right) +1\right) }{\delta _1 \left( c_2 e^{c_1 (\xi +\chi )}+1\right) ^2}\right) ,~c_1<0. \end{aligned}$$

**CASE 3:**    $$c_1=0,~~c_3=0$$,

**FAMILY-I**

**Figure 2:**
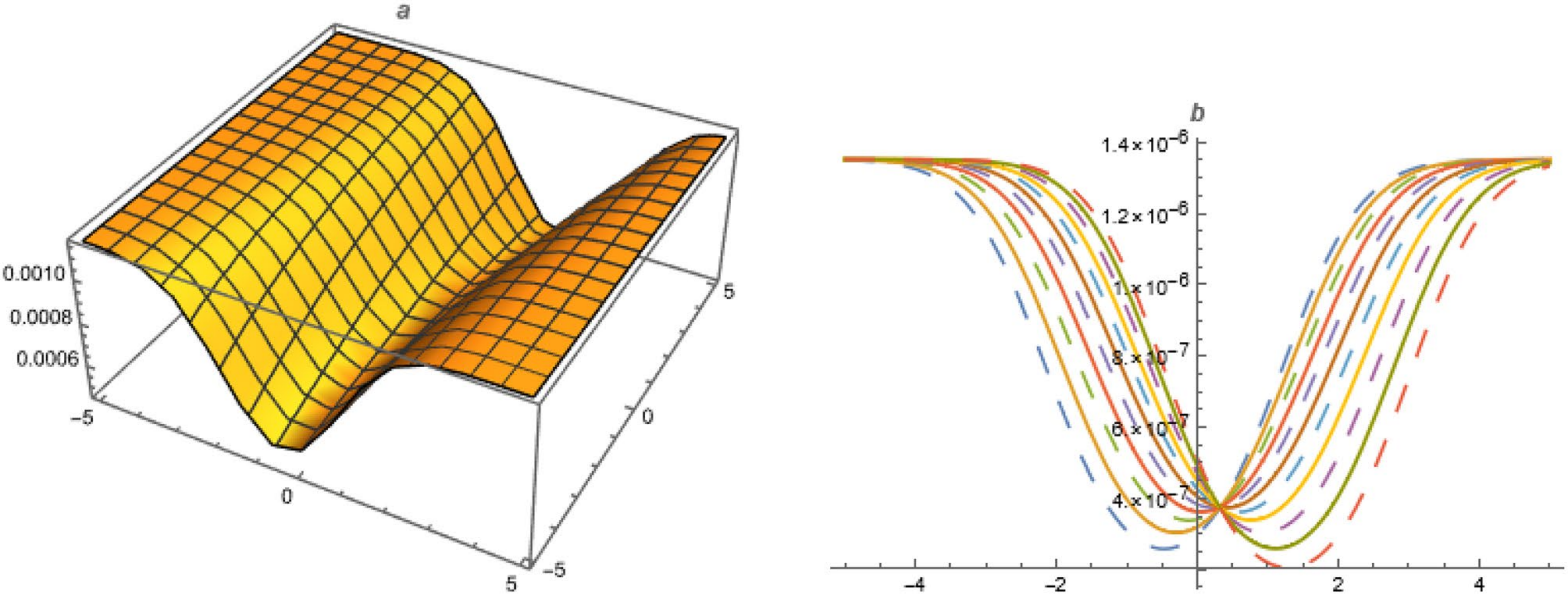
Solution $$U_{3}$$ with $$\alpha =1.1,~\beta =1.1,~c_1=0.1,~c_2=-0.1,~\gamma =-2,~\delta _1=-1,~\delta _2=3.3,~\delta _3=-0.01,~\delta _4=0.03,~\delta _5=0.1,~k=-3.1,~\mu =1,~\chi =-1.1,~y=1,~z=1.$$.

46 $$\begin{aligned}&A_0=-\frac{2 \alpha ^2 c_0 c_2 \delta _3 k^2}{\delta _1},~A_{-1}=A_1=A_{-2}=0,~A_2=-\frac{6 \alpha ^2 c_2^2 \delta _3 k^2}{\delta _1},~\omega =\frac{\alpha ^2 \delta _3+\beta ^2 \delta _4+\gamma ^2 \delta _5+4 \alpha ^4 c_0 c_2 \delta _3 k^2}{\alpha } \end{aligned}$$

Put (46) in (38),

47 $$\begin{aligned}&U_{5}=\left( -\frac{\left( 6 \alpha ^2 c_2^2 \delta _3 k^2\right) \left( \frac{\sqrt{c_0 c_2} \tan \left( \sqrt{k_0 k_2} (\xi +\chi )\right) }{c_2}\right) ^2}{\delta _1}-\frac{2 \alpha ^2 c_0 c_2 \delta _3 k^2}{\delta _1}\right) ,~~c_0c_2>0, \end{aligned}$$

48 $$\begin{aligned}&U_{6}=\left( -\frac{\left( 6 \alpha ^2 c_2^2 \delta _3 k^2\right) \left( -\frac{\sqrt{-c_0 c_2} \tanh \left( \sqrt{-c_0 c_2} (\xi +\chi )\right) }{c_2}\right) ^2}{\delta _1}-\frac{2 \alpha ^2 c_0 c_2 \delta _3 k^2}{\delta _1}\right) ,~~c_0c_2<0. \end{aligned}$$

**FAMILY-II**

49 $$\begin{aligned}&A_0=-\frac{6 \alpha ^2 c_0 c_2 \delta _3 k^2}{\delta _1},~A_{-1}=A_1=A_2=0,~A_{-2}=-\frac{6 \alpha ^2 c_0^2 \delta _3 k^2}{\delta _1},~\omega =\frac{\alpha ^2 \delta _3+\beta ^2 \delta _4+\gamma ^2 \delta _5-4 \alpha ^4 c_0 c_2 \delta _3 k^2}{\alpha } \end{aligned}$$

Put (49) in (38),

50 $$\begin{aligned}&U_{7}=\left( -\frac{\left( 6 \alpha ^2 c_0^2 \delta _3 k^2\right) \left( \frac{1}{\left( \frac{\sqrt{c_0 c_2} \tan \left( \sqrt{c_0 c_2} (\xi +\chi )\right) }{c_2}\right) ^2}\right) ^2}{\delta _1}-\frac{6 \alpha ^2 c_0 c_2 \delta _3 k^2}{\delta _1}\right) ,~c_0c_2>0, \end{aligned}$$

51 $$\begin{aligned}&U_{8}=\left( -\frac{\left( \frac{1}{\left( \frac{\sqrt{-k_0 k_2} \tanh \left( \sqrt{-k_0 k_2} \left( \xi +\xi _0\right) \right) }{k_2}\right) ^2}\right) ^2 \left( 6 \alpha ^2 c_0^2 \delta _3 k^2\right) }{\delta _1}-\frac{6 \alpha ^2 c_0 c_2 \delta _3 k^2}{\delta _1}\right) ,~c_0c_2<0. \end{aligned}$$

**FAMILY-III**

52 $$\begin{aligned}&A_0=-\frac{12 \alpha ^2 c_0 c_2 \delta _3 k^2}{\delta _1},~A_{-1}=A_1=0,~A_{-2}=-\frac{6 \alpha ^2 c_0^2 \delta _3 k^2}{\delta _1},~\nonumber \\ {}&A_2=-\frac{6 \alpha ^2 c_2^2 \delta _3 k^2}{\delta _1},~\omega =\frac{\alpha ^2 \delta _3+\beta ^2 \delta _4+\gamma ^2 \delta _5-16 \alpha ^4 c_0 c_2 \delta _3 k^2}{\alpha } \end{aligned}$$

Put (52) in (38),

53 $$\begin{aligned}&U_{9}=\left( -\frac{6 \alpha ^2 c_0 c_2 \delta _3 k^2 \left( \csc ^2\left( \sqrt{c_0 c_2} (\xi +\chi )\right) +\sec ^2\left( \sqrt{k_0 k_2} (\xi +\chi )\right) \right) }{\delta _1}\right) ,~c_0c_2>0, \end{aligned}$$

54 $$\begin{aligned}&U_{10}=\left( \frac{24 \alpha ^2 c_0 c_2 \delta _3 k^2 \text {csch}^2\left( 2 \sqrt{-c_0 c_2} (\xi +\chi )\right) }{\delta _1}\right) ,~c_0c_2<0. \end{aligned}$$

### Application of modified extended auxiliary equation mapping method

Let Eq. (37) has solution as;

55 $$\begin{aligned} U=A_2 \Psi ^2+A_1 \Psi +A_0+\frac{B_1}{\Psi }+\frac{B_2}{\Psi ^2}+C_2 \Psi '+D_2 \left( \frac{\Psi '}{\Psi }\right) ^2+D_1\left( \frac{ \Psi '}{\Psi }\right) \end{aligned}$$

Put (55) with (17) in (37),

56 $$\begin{aligned}&A_0=\frac{\alpha ^2 \beta _1 \delta _3 \left( -k^2\right) -\beta _1 \delta _1 D_2}{\delta _1},~A_1=\frac{-2 \beta _2 \delta _1 D_2-3 \alpha ^2 \beta _2 \delta _3 k^2}{2 \delta _1},~A_2=\frac{-\beta _3 \delta _1 D_2-3 \alpha ^2 \beta _3 \delta _3 k^2}{\delta _1},\nonumber \\ {}&C_2=\frac{3 \alpha ^2 \sqrt{\beta _3} \delta _3 k^2}{\delta _1},~B_1=0,~B_2=0,~D_1=0,~\omega =\frac{\alpha ^2 \delta _3+\beta ^2 \delta _4+\gamma ^2 \delta _5+\alpha ^4 \beta _1 \delta _3 \left( -k^2\right) }{\alpha } \end{aligned}$$

Put (56) in (55)

**Figure 3:**
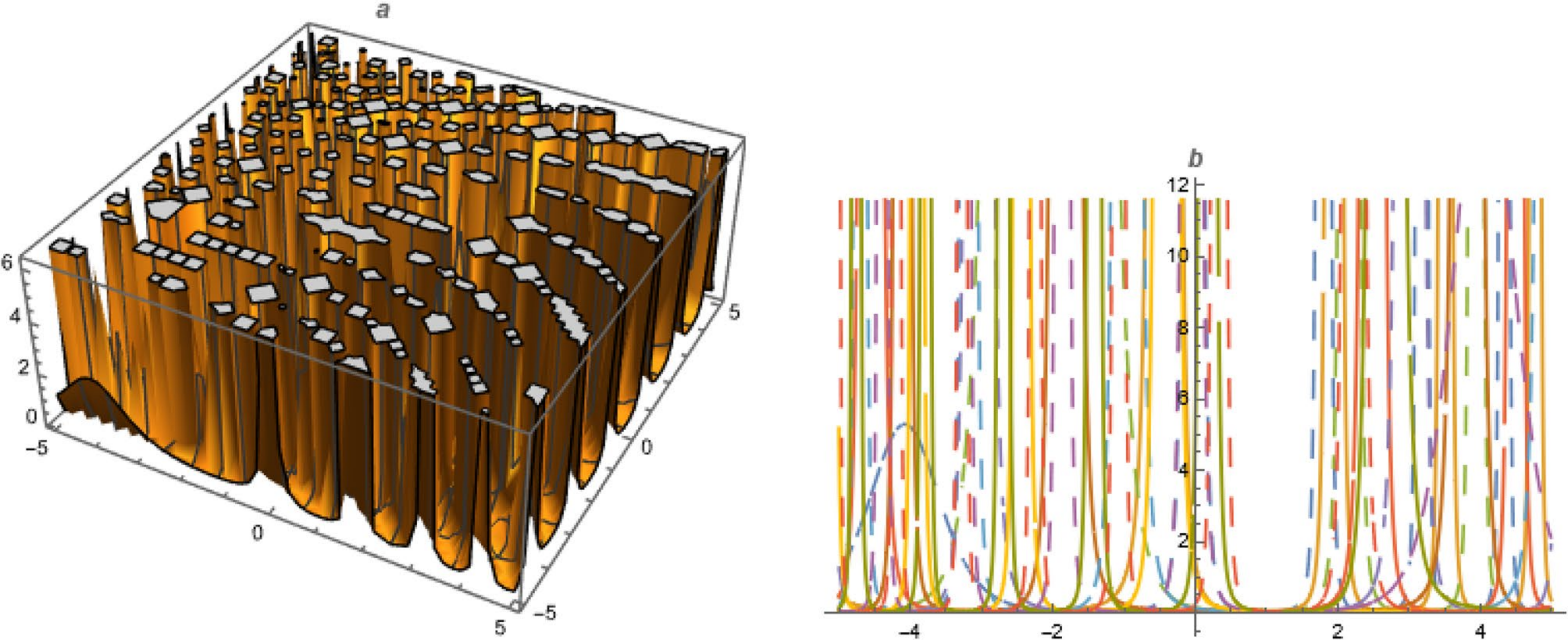
Solution $$U_{10}$$ with $$\alpha =-1,~\beta =1,~c_0=1.5,~c_2=-0.5,~\gamma =1,~\delta _1=1,~\delta _2=1.3,~\delta _3=1.1,~\delta _4=0.3,~\delta _5=0.5,~k=-0.1,~\mu =1,~\chi =-0.7,~y=1,~z=1.$$.

**CASE I**:

57 $$\begin{aligned} U_{11}&=\left( \frac{\alpha ^2 \beta _1 \delta _3 \left( -k^2\right) -\beta _1 \delta _1 D_2}{\delta _1}-\frac{\left( \beta _1 \left( \epsilon \coth \left( \frac{1}{2} \sqrt{\beta _1} (\xi +\chi )\right) +1\right) \right) \left( -2 \beta _2 \delta _1 D_2-3 \alpha ^2 \beta _2 \delta _3 k^2\right) }{\beta _2 \left( 2 \delta _1\right) }\right) \nonumber \\ {}&\quad +\left( \frac{\left( -\frac{\beta _1 \left( \epsilon \coth \left( \frac{1}{2} \sqrt{\beta _1} (\xi +\chi )\right) +1\right) }{\beta _2}\right) ^2 \left( -\beta _3 \delta _1 D_2-3 \alpha ^2 \beta _3 \delta _3 k^2\right) }{\delta _1}\right) +D_2 \left( \frac{\beta _1^{3/2} \epsilon \text {csch}^2\left( \frac{1}{2} \sqrt{\beta _1} (\xi +\chi )\right) }{\frac{\left( 2 \beta _2\right) \left( -\beta _1 \left( \epsilon \coth \left( \frac{1}{2} \sqrt{\beta _1} (\xi +\chi )\right) +1\right) \right) }{\beta _2}}\right) ^2\nonumber \\ {}&\quad +\left( \frac{\left( +\frac{3 \alpha ^2 \sqrt{\beta _3} \delta _3 k^2}{\delta _1}\right) \left( \beta _1^{3/2} \epsilon \text {csch}^2\left( \frac{1}{2} \sqrt{\beta _1} (\xi +\chi )\right) \right) }{2 \beta _2}\right) ,~~\beta _1>0,~~\beta _2^2-4\beta _1\beta _3=0. \end{aligned}$$

**CASE II**:

58 $$\begin{aligned} U_{12}&=\left( \frac{\alpha ^2 \beta _1 \delta _3 \left( -k^2\right) -\beta _1 \delta _1 D_2}{\delta _1}+\frac{\left( -2 \beta _2 \delta _1 D_2-3 \alpha ^2 \beta _2 \delta _3 k^2\right) \left( -\sqrt{\frac{\beta _1}{4 \beta _3}} \left( \frac{\epsilon \sinh \left( \sqrt{\beta _1} (\xi +\chi )\right) }{\cosh \left( \sqrt{\beta _1} (\xi +\chi )\right) +\eta }+1\right) \right) }{2 \delta _1}\right) +\nonumber \\ {}&\quad \times \left( \frac{\left( -\beta _3 \delta _1 D_2-3 \alpha ^2 \beta _3 \delta _3 k^2\right) \left( -\sqrt{\frac{\beta _1}{4 \beta _3}} \left( \frac{\epsilon \sinh \left( \sqrt{\beta _1} (\xi +\chi )\right) }{\cosh \left( \sqrt{\beta _1} (\xi +\chi )\right) +\eta }+1\right) \right) ^2}{\delta _1}\right) +\left( \frac{3 \alpha ^2 \sqrt{\beta _3} \delta _3 k^2}{\delta _1}\right) \nonumber \\ {}&\quad \times \left( -\frac{1}{2} \sqrt{\frac{\beta _1}{\beta _3}} \left( \frac{\sqrt{\beta _1} \epsilon \cosh \left( \sqrt{\beta _1} (\xi +\chi )\right) }{\cosh \left( \sqrt{\beta _1} (\xi +\chi )\right) +\eta }-\frac{\sqrt{\beta _1} \epsilon \sinh ^2\left( \sqrt{\beta _1} (\xi +\chi )\right) }{\left( \cosh \left( \sqrt{\beta _1} (\xi +\chi )\right) +\eta \right) ^2}\right) \right) +D_2\left( \Omega \right) ,~\beta _1>0,~\beta _3>0, ~\beta _2=\left( 4\beta _1\beta _3\right) ^{1/2} \end{aligned}$$

where $$\Omega = \left( -\frac{\sqrt{\frac{\beta _1}{\beta _3}} \left( \frac{\sqrt{\beta _1} \epsilon \cosh \left( \sqrt{\beta _1} (\xi +\chi )\right) }{\cosh \left( \sqrt{\beta _1} (\xi +\chi )\right) +\eta }-\frac{\sqrt{\beta _1} \epsilon \sinh ^2\left( \sqrt{\beta _1} (\xi +\chi )\right) }{\left( \cosh \left( \sqrt{\beta _1} (\xi +\chi )\right) +\eta \right) {}^2}\right) }{2 \left( -\sqrt{\frac{\beta _1}{4 \beta _3}} \left( \frac{\epsilon \sinh \left( \sqrt{\beta _1} (\xi +\chi )\right) }{\cosh \left( \sqrt{\beta _1} (\xi +\chi )\right) +\eta }+1\right) \right) }\right) ^2$$

**CASE III**:

59 $$\begin{aligned} U_{13}&=\left( \frac{\alpha ^2 \beta _1 \delta _3 \left( -k^2\right) -\beta _1 \delta _1 D_2}{\delta _1}\right) +D_2 \left( -\frac{\beta _1 \left( \frac{\sqrt{\beta _1} \epsilon \cosh \left( \sqrt{\beta _1} (\xi +\chi )\right) }{\cosh \left( \sqrt{\beta _1} (\xi +\chi )\right) +\eta \sqrt{P^2+1}}-\frac{\sqrt{\beta _1} \epsilon \sinh \left( \sqrt{\beta _1} (\xi +\chi )\right) \left( \sinh \left( \sqrt{\beta _1} (\xi +\chi )\right) +P\right) }{\left( \cosh \left( \sqrt{\beta _1} (\xi +\chi )\right) +\eta \sqrt{P^2+1}\right) ^2}\right) }{\frac{\beta _2 \left( -\beta _1 \left( \frac{\epsilon \left( \sinh \left( \sqrt{\beta _1} (\xi +\chi )\right) +P\right) }{\cosh \left( \sqrt{\beta _1} (\xi +\chi )\right) +\eta \sqrt{P^2+1}}+1\right) \right) }{\beta _2}}\right) ^2\nonumber \\ {}&\quad \times \left( \frac{\left( -2 \beta _2 \delta _1 D_2-3 \alpha ^2 \beta _2 \delta _3 k^2\right) \left( -\beta _1 \left( \frac{\epsilon \left( \sinh \left( \sqrt{\beta _1} (\xi +\chi )\right) +P\right) }{\cosh \left( \sqrt{\beta _1} (\xi +\chi )\right) +\eta \sqrt{P^2+1}}+1\right) \right) }{\beta _2 \left( 2 \delta _1\right) }\right) +\left( \frac{3 \alpha ^2 \sqrt{\beta _3} \delta _3 k^2}{\delta _1}\right) \nonumber \\ {}&\quad \times \left( -\frac{\beta _1 \left( \frac{\sqrt{\beta _1} \epsilon \cosh \left( \sqrt{\beta _1} (\xi +\chi )\right) }{\cosh \left( \sqrt{\beta _1} (\xi +\chi )\right) +\eta \sqrt{P^2+1}}-\frac{\sqrt{\beta _1} \epsilon \sinh \left( \sqrt{\beta _1} (\xi +\chi )\right) \left( \sinh \left( \sqrt{\beta _1} (\xi +\chi )\right) +P\right) }{\left( \cosh \left( \sqrt{\beta _1} (\xi +\chi )\right) +\eta \sqrt{P^2+1}\right) ^2}\right) }{\beta _2}\right) +\left( \frac{-\beta _3 \delta _1 D_2-3 \alpha ^2 \beta _3 \delta _3 k^2}{\delta _1}\right) \nonumber \\ {}&\quad \times \left( \left( -\frac{\beta _1 \left( \frac{\epsilon \left( \sinh \left( \sqrt{\beta _1} (\xi +\chi )\right) +P\right) }{\cosh \left( \sqrt{\beta _1} (\xi +\chi )\right) +\eta \sqrt{P^2+1}}+1\right) }{\beta _2}\right) ^2\right) ,~~\beta _1>0. \end{aligned}$$

### Application of $G'/G$-expansion method

Let Eq. (37) has solution as;

60 $$\begin{aligned} U=A_1\left( \frac{ G'}{G}\right) +A_2\left( \frac{ G'^2}{G^2}\right) +A_0 \end{aligned}$$

Put (60) with (22) in (37),

61 $$\begin{aligned}&A_0=-\frac{\alpha ^2 \delta _3 k^2 \left( \lambda _1^2+2 \mu _1\right) }{\delta _1},~A_1=-\frac{6 \alpha ^2 \delta _3 k^2 \lambda _1}{\delta _1},~A_2=-\frac{6 \alpha ^2 \delta _3 k^2}{\delta _1},\nonumber \\ {}&\omega =\frac{\alpha ^2 \delta _3+\beta ^2 \delta _4+\gamma ^2 \delta _5+\alpha ^4 \delta _3 \left( -k^2\right) \lambda _1^2+4 \alpha ^4 \delta _3 k^2 \mu _1}{\alpha } \end{aligned}$$

Put (61) in (60),

**Figure 4:**
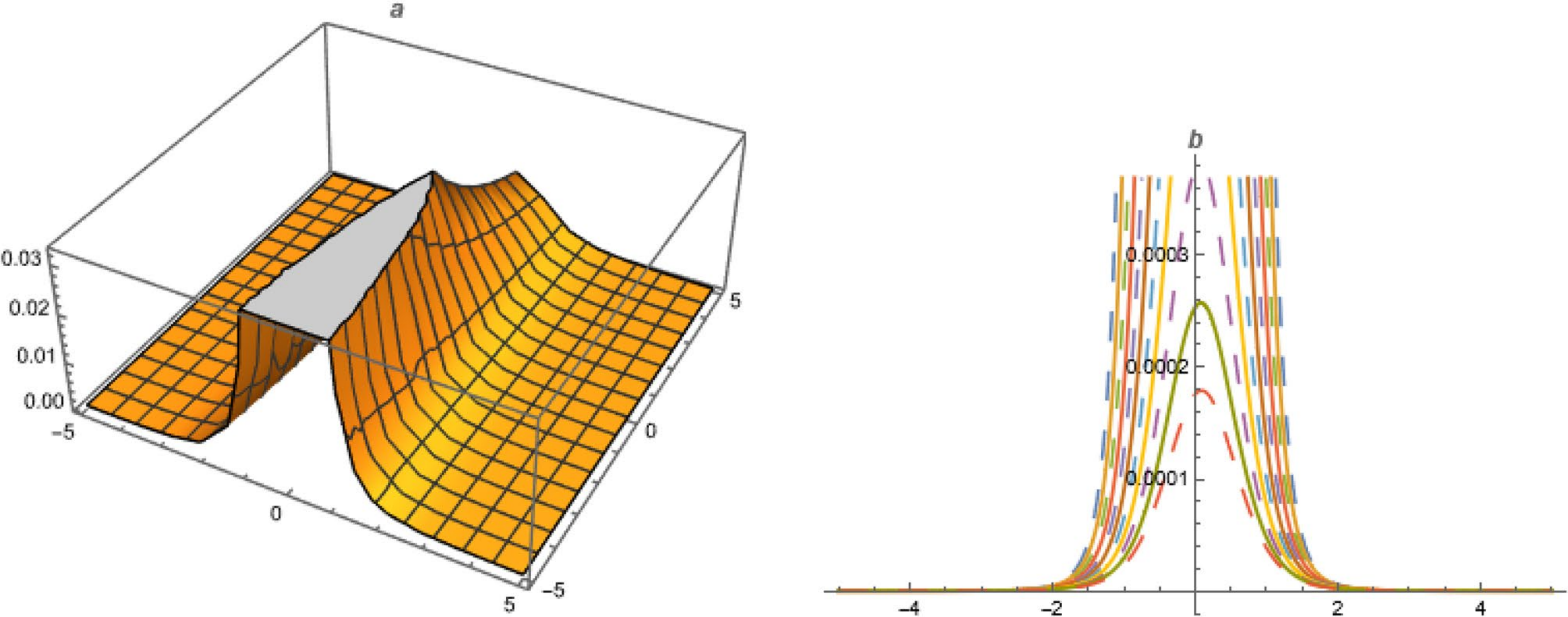
Solution $$U_{11}$$ with $$\alpha =-1,~\beta _1=\beta _3=1,~\beta _2=2,~\beta =0.03,~\gamma =0.07,~\delta _1=1.01,~\delta _2=-1.3,~\delta _3=0.007,~\delta _4=0.3,~\delta _5=1.5,~D_2=-0.03,~k=-0.1,~\mu =1,~\chi =0.1,~y=1,~z=1,~\epsilon =-1.$$.

**CASE I**:          $$\lambda _1^2-4 \mu _1 >0$$

62 $$\begin{aligned} U_{14}&=\left( -\left( \alpha ^2 \delta _3 k^2 \left( \lambda _1^2-4 \mu _1\right) \left( 2 P_2 P_1 \sinh \left( \sqrt{\lambda _1^2-4 \mu _1}\right) +\left( P_1^2+P_2^2\right) \cosh \left( \sqrt{\lambda _1^2-4 \mu _1}\right) -2 P_1^2+2 P_2^2\right) \right. \right) \nonumber \\ {}&\quad /\left. 2 \delta _1 \left( P_2 \sinh \left( \frac{1}{2} \sqrt{\lambda _1^2-4 \mu _1}\right) +P_1 \cosh \left( \frac{1}{2} \sqrt{\lambda _1^2-4 \mu _1}\right) \right) ^2\right) \end{aligned}$$

**CASE II**:          $$\lambda _1 ^2-4 \mu _1<0$$

63 $$\begin{aligned} U_{15}&=\left( -\left( \alpha ^2 \delta _3 k^2 \left( \lambda _1^2-4 \mu _1\right) \left( 2 P_1 P_2 \sin \left( \sqrt{4 \mu _1-\lambda _1^2}\right) +\left( P_1^2-P_2^2\right) \cos \left( \sqrt{4 \mu _1-\lambda _1^2}\right) -2 \left( P_1^2+P_2^2\right) \right) \right. \right) \nonumber \\ {}&\quad /\left. \left. 2 \delta _1 \left( P_2 \sin \left( \frac{1}{2} \sqrt{4 \mu _1-\lambda _1^2}\right) +P_1 \cos \left( \frac{1}{2} \sqrt{4 \mu _1-\lambda _1^2}\right) \right) ^2\right) \right) \end{aligned}$$

**CASE III**:          $$\lambda _1 ^2-4 \mu _1=0$$

64 $$\begin{aligned} U_{16}&=-\left( \frac{\alpha ^2 \delta _3 k^2 \left( \lambda _1^2+2 \mu _1\right) }{\delta _1}\right) -\left( \frac{\left( \frac{P_2}{\xi \text {P}_2+P_1}-\frac{\lambda _1}{2}\right) \left( 6 \alpha ^2 \delta _3 k^2 \lambda _1\right) }{\delta _1}\right) \nonumber \\ {}&\quad -\left( \frac{\left( 6 \alpha ^2 \delta _3 k^2\right) \left( \frac{P_2}{\xi \text {P}_2+P_1}-\frac{\lambda _1}{2}\right) ^2}{\delta _1}\right) \end{aligned}$$

### Application of $Exp(-\Psi (\xi ))$-expansion method

Let Eq. (37) has solution as;

65 $$\begin{aligned} U=A_1 \exp (-\Psi (\xi ))+A_2 \exp ( -2\Psi (\xi ))+A_0 \end{aligned}$$

Put (65) with (27) in (37),

66 $$\begin{aligned}&A_1=-\frac{6 \alpha ^2 \delta _3 k^2 \lambda _1}{\delta _1},~A_0=-\frac{\alpha ^2 \delta _3 k^2 \left( \lambda _1^2+2 \mu _1\right) }{\delta _1},~A_2=-\frac{6 \alpha ^2 \delta _3 k^2}{\delta _1},\nonumber \\ {}&~\omega =\frac{\alpha ^2 \delta _3+\beta ^2 \delta _4+\gamma ^2 \delta _5+\alpha ^4 \delta _3 \left( -k^2\right) \lambda _1^2+4 \alpha ^4 \delta _3 k^2 \mu _1}{\alpha } \end{aligned}$$

Putting (66) with (65),

**CASE I**:          $$\lambda _1^2-4 \mu _1 >0$$,   $$\mu _1\ne 0$$

67 $$\begin{aligned} U_{17}&=\left( -\frac{\left( 6 \alpha ^2 \delta _3 k^2\right) \log ^2\left( \frac{-\sqrt{\lambda _1^2-4 \mu _1} \tanh \left( \frac{1}{2} \sqrt{\lambda _1^2-4 \mu _1} (\xi +\chi )\right) -\lambda _1}{2 \mu _1}\right) }{\delta _1}\right) \nonumber \\ {}&\quad -\left( \frac{\left( 6 \alpha ^2 \delta _3 k^2 \lambda _1\right) \log \left( \frac{-\sqrt{\lambda _1^2-4 \mu _1} \tanh \left( \frac{1}{2} \sqrt{\lambda _1^2-4 \mu _1} (\xi +\chi )\right) -\lambda _1}{2 \mu _1}\right) }{\delta _1}\right) \nonumber \\ {}&\quad -\left( \frac{\alpha ^2 \delta _3 k^2 \left( \lambda _1^2+2 \mu _1\right) }{\delta _1}\right) \end{aligned}$$

**CASE II**:          $$\lambda _1^2-4 \mu _1 >0$$,  $$\mu _1=0$$

68 $$\begin{aligned} U_{18}=\left( -\frac{\left( -\log \left( \frac{\lambda }{e^{\lambda \left( \xi +\chi \right) }-1}\right) \right) \left( 6 \alpha ^2 \delta _3 k^2 \lambda _1\right) }{\delta _1}+\frac{\left( -\left( 6 \alpha ^2 \delta _3 k^2\right) \right) \left( -\log \left( \frac{\lambda }{e^{\lambda \left( \xi +\chi \right) }-1}\right) \right) ^2}{\delta _1}-\frac{\alpha ^2 \delta _3 k^2 \lambda _1^2}{\delta _1}\right) \end{aligned}$$

**CASE III**:        $$\lambda _1^2-4 \mu _1=0$$,  $$\mu _1,~\lambda _1\ne 0$$

69 $$\begin{aligned} U_{19}=-\left( \frac{\alpha ^2 \delta _3 k^2 \left( \lambda _1^2+2 \mu _1\right) }{\delta _1}\right) -\left( \frac{\left( 6 \alpha ^2 \delta _3 k^2 \lambda _1\right) \log \left( \frac{2-2 \lambda _1 (\xi +\chi )}{\lambda _1^2 (\xi +\chi )}\right) }{\delta _1}\right) +\left( \frac{\left( -\left( 6 \alpha ^2 \delta _3 k^2\right) \right) \log \left( \frac{2-2 \lambda _1 (\xi +\chi )}{\lambda _1^2 (\xi +\chi )}\right) ^2}{\delta _1}\right) \end{aligned}$$

**CASE IV**:          $$\lambda _1^2-4 \mu =0$$, $$\mu _1=\lambda _1=0$$

70 $$\begin{aligned} U_{20}=-\left( \frac{\left( 6 \alpha ^2 \delta _3 k^2\right) \log (\xi +\chi )^2}{\delta _1}\right) \end{aligned}$$

**CASE V**:          $$\lambda _1^2-4 \mu _1 <0$$

71 $$\begin{aligned} U_{21}&=\left( -\frac{\left( 6 \alpha ^2 \delta _3 k^2\right) \log ^2\left( -\frac{\sqrt{4 \mu _1-\lambda _1^2} \tan \left( \frac{1}{2} \sqrt{4 \mu _1-\lambda _1^2} (\xi +\chi )\right) +\lambda _1}{2 \mu _1}\right) }{\delta _1}\right) \nonumber \\ {}&\quad -\left( \frac{\left( 6 \alpha ^2 \delta _3 k^2 \lambda _1\right) \log \left( -\frac{\sqrt{4 \mu _1-\lambda _1^2} \tan \left( \frac{1}{2} \sqrt{4 \mu _1-\lambda _1^2} (\xi +\chi )\right) +\lambda _1}{2 \mu _1}\right) }{\delta _1}\right) \nonumber \\ {}&-\quad \left( \frac{\alpha ^2 \delta _3 k^2 \left( \lambda _1^2+2 \mu _1\right) }{\delta _1}\right) \end{aligned}$$

### Application of modified F-expansion method

Let Eq. (37) has as;

72 $$\begin{aligned} U=a_2 F^2+a_1 F+a_0+\frac{b_2}{F^2}+\frac{b_1}{F} \end{aligned}$$

Put (72) with (34) in (37),

**For A = 0, B = 1, C = −1, **

73 $$\begin{aligned}&a_0=-\frac{\alpha ^2 \delta _3 k^2}{\delta _1},~a_2=-\frac{6 \alpha ^2 \delta _3 k^2}{\delta _1},~a_1=\frac{6 \alpha ^2 \delta _3 k^2}{\delta _1},\nonumber \\ {}&b_1=0,~b_2=0,~\omega =\frac{\alpha ^2 \delta _3+\beta ^2 \delta _4+\gamma ^2 \delta _5+\alpha ^4 \delta _3 \left( -k^2\right) }{\alpha } \end{aligned}$$

Put (73) in (72),

74 $$\begin{aligned} U_{22}=-\left( \frac{\alpha ^2 \delta _3 k^2}{\delta _1}\right) +\left( \frac{6 \alpha ^2 \delta _3 k^2}{\delta _1}\right) \left( \frac{1}{2} \tanh \left( \frac{\xi }{2}\right) +\frac{1}{2}\right) -\left( \frac{6 \alpha ^2 \delta _3 k^2}{\delta _1}\right) \left( \left( \frac{1}{2} \tanh \left( \frac{\xi }{2}\right) +\frac{1}{2}\right) ^2\right) \end{aligned}$$

**For A=0, C=1, B=-1, **

75 $$\begin{aligned}&a_0=-\frac{\alpha ^2 \delta _3 k^2}{\delta _1},~a_2=-\frac{6 \alpha ^2 \delta _3 k^2}{\delta _1},~a_1=\frac{6 \alpha ^2 \delta _3 k^2}{\delta _1},\nonumber \\ {}&b_1=b_2=0,~\omega =\frac{\alpha ^2 \delta _3+\beta ^2 \delta _4+\gamma ^2 \delta _5+\alpha ^4 \delta _3 \left( -k^2\right) }{\alpha } \end{aligned}$$

**Figure 5:**
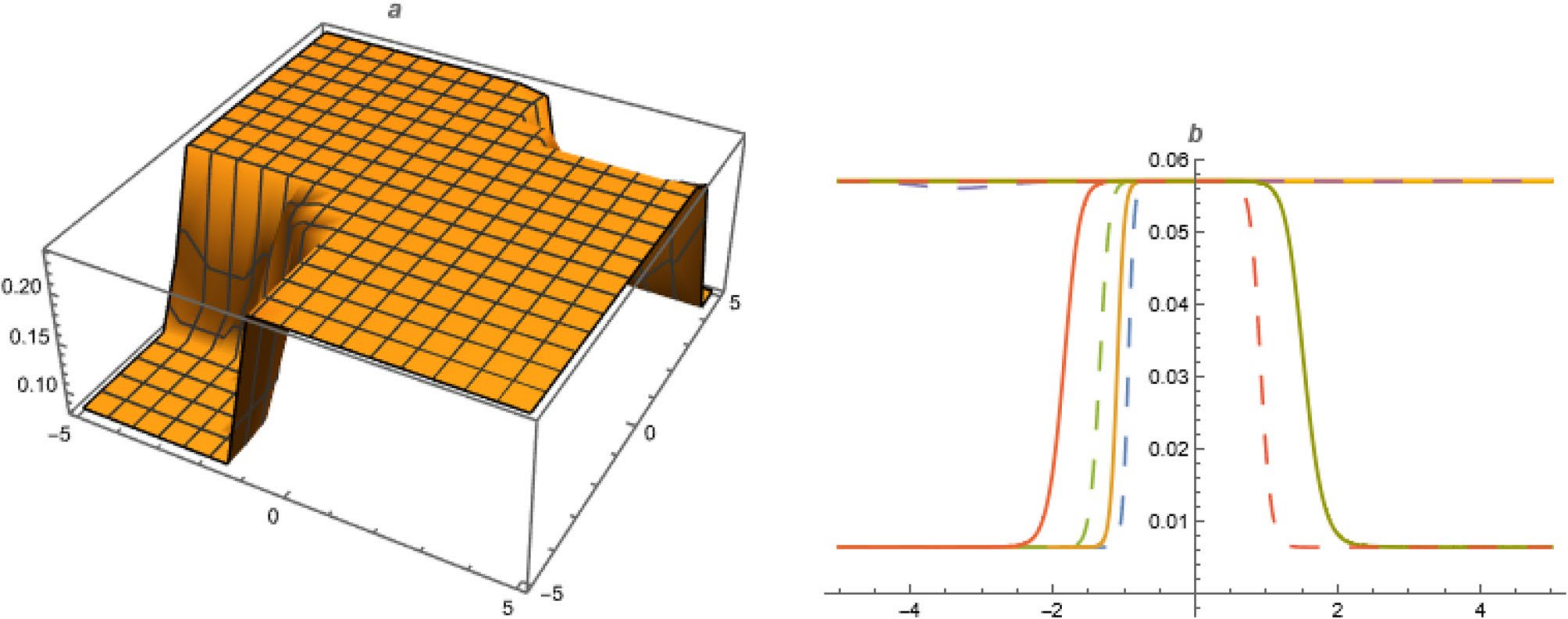
Solution $$U_{17}$$ with $$\alpha =0.1,~\beta =0.3,~\gamma =0.07,~\delta _1=1.01,~\delta _2=-1.3,~\delta _3=0.007,~\delta _4=0.3,~\delta _5=0.5,~\lambda _1=4,~k=4.1,~\mu _1=0.2,~\mu =1,~\chi =5.1,~y=1,~z=1.$$.

Put (75) into (72),

76 $$\begin{aligned} U_{23}=\left( -\frac{\alpha ^2 \delta _3 k^2}{\delta _1}\right) +\left( \frac{6 \alpha ^2 \delta _3 k^2}{\delta _1}\right) \left( \frac{1}{2}-\frac{1}{2} \coth \left( \frac{\xi }{2}\right) \right) -\frac{6 \alpha ^2 \delta _3 k^2}{\delta _1}\left( \frac{1}{2}-\frac{1}{2} \coth \left( \frac{\xi }{2}\right) \right) ^2 \end{aligned}$$

**For A=1/2, B=0, C=-1/2 **

**FAMILY-I**

77 $$\begin{aligned} a_0=\frac{3 \alpha ^2 \delta _3 k^2}{2 \delta _1},~a_2=-\frac{3 \alpha ^2 \delta _3 k^2}{2 \delta _1},~a_1=~b_1=b_2=0,~\omega =\frac{\alpha ^2 \delta _3+\beta ^2 \delta _4+\gamma ^2 \delta _5+\alpha ^4 \delta _3 k^2}{\alpha } \end{aligned}$$

Substitute (77) into (72),

78 $$\begin{aligned} U_{24,1}=\frac{3 \alpha ^2 \delta _3 k^2}{2 \delta _1}-\left( \frac{3 \alpha ^2 \delta _3 k^2}{2 \delta _1}\right) (\coth (\xi )+\text {csch}(\xi ))^2 \end{aligned}$$

**FAMILY-II**

79 $$\begin{aligned} a_0=\frac{3 \alpha ^2 \delta _3 k^2}{2 \delta _1},~a_2=0,~a_1=0,~b_1=0,~b_2=-\frac{3 \alpha ^2 \delta _3 k^2}{2 \delta _1}~,\omega =\frac{\alpha ^2 \delta _3+\beta ^2 \delta _4+\gamma ^2 \delta _5+\alpha ^4 \delta _3 k^2}{\alpha } \end{aligned}$$

Put (79) in (72),

80 $$\begin{aligned} U_{24,2}=\frac{3 \alpha ^2 \delta _3 k^2}{2 \delta _1}-\frac{3 \alpha ^2 \delta _3 k^2}{2 \delta _1}\left( \frac{1}{(\coth (\xi )+\text {csch}(\xi ))^2}\right) \end{aligned}$$

**FAMILY-III**

81 $$\begin{aligned}&a_0=-\frac{\alpha ^2 \delta _3 k^2}{\delta _1},~a_2=-\frac{3 \alpha ^2 \delta _3 k^2}{2 \delta _1},~a_1=0,~b_1=0,~\nonumber \\ {}&b_2=-\frac{3 \alpha ^2 \delta _3 k^2}{2 \delta _1},~\omega =\frac{\alpha ^2 \delta _3+\beta ^2 \delta _4+\gamma ^2 \delta _5-4 \alpha ^4 \delta _3 k^2}{\alpha } \end{aligned}$$

Put (81) in (72),

82 $$\begin{aligned} U_{24,3}=-\frac{\alpha ^2 \delta _3 k^2}{\delta _1}-\frac{3 \alpha ^2 \delta _3 k^2}{2 \delta _1}\left( (\coth (\xi )+\text {csch}(\xi ))^2\right) -\frac{3 \alpha ^2 \delta _3 k^2}{2 \delta _1} \left( \frac{1}{(\coth (\xi )+\text {csch}(\xi ))^2}\right) \end{aligned}$$

**For A=1, B=0, C=-1 **

**FAMILY-I**

83 $$\begin{aligned} a_0=\frac{6 \alpha ^2 \delta _3 k^2}{\delta _1},~a_2=-\frac{6 \alpha ^2 \delta _3 k^2}{\delta _1},~a_1=b_1=b_2=0,~\omega =\frac{\alpha ^2 \delta _3+\beta ^2 \delta _4+\gamma ^2 \delta _5+4 \alpha ^4 \delta _3 k^2}{\alpha } \end{aligned}$$

Put (83) in (72),

84 $$\begin{aligned} U_{25,1}=\left( \frac{6 \alpha ^2 \delta _3 k^2}{\delta _1}\right) -\left( \frac{6 \alpha ^2 \delta _3 k^2}{\delta _1}\right) \left( \tanh ^2(\xi )\right) \end{aligned}$$

**FAMILY-II**

85 $$\begin{aligned} a_0=\frac{6 \alpha ^2 \delta _3 k^2}{\delta _1},~a_2=0,~a_1=0,~b_1=0,~b_2=-\frac{6 \alpha ^2 \delta _3 k^2}{\delta _1},~\omega =\frac{\alpha ^2 \delta _3+\beta ^2 \delta _4+\gamma ^2 \delta _5+4 \alpha ^4 \delta _3 k^2}{\alpha } \end{aligned}$$

Put (85) in (72),

86 $$\begin{aligned} U_{25,2}=\left( \frac{6 \alpha ^2 \delta _3 k^2}{\delta _1}\right) -\left( \frac{6 \alpha ^2 \delta _3 k^2}{\delta _1}\right) \left( \frac{1}{\tanh ^2(\xi )}\right) \end{aligned}$$

**FAMILY-III**

87 $$\begin{aligned} a_0=-\frac{4 \alpha ^2 \delta _3 k^2}{\delta _1},~a_2=-\frac{6 \alpha ^2 \delta _3 k^2}{\delta _1},~a_1=0,~b_1=0,~b_2=-\frac{6 \alpha ^2 \delta _3 k^2}{\delta _1},~\omega =\frac{\alpha ^2 \delta _3+\beta ^2 \delta _4+\gamma ^2 \delta _5-16 \alpha ^4 \delta _3 k^2}{\alpha } \end{aligned}$$

Put (87) in (72),

88 $$\begin{aligned} U_{25,3}=\left( -\frac{4 \alpha ^2 \delta _3 k^2}{\delta _1}\right) -\left( \frac{6 \alpha ^2 \delta _3 k^2}{\delta _1}\right) \left( \tanh (\xi )^2\right) -\left( \frac{6 \alpha ^2 \delta _3 k^2}{\delta _1}\right) \left( \frac{1}{\tanh ^2(\xi )}\right) \end{aligned}$$

** For A=C=1/2, B=0, **

**FAMILY-I**

89 $$\begin{aligned} a_0=-\frac{\alpha ^2 \delta _3 k^2}{2 \delta _1},~a_2=-\frac{3 \alpha ^2 \delta _3 k^2}{2 \delta _1},~a_1=0,~b_1=0,~b_2=0,~\omega =\frac{\alpha ^2 \delta _3+\beta ^2 \delta _4+\gamma ^2 \delta _5+\alpha ^4 \delta _3 k^2}{\alpha } \end{aligned}$$

Put (89) in (72),

90 $$\begin{aligned} U_{26,1}=\left( -\frac{\alpha ^2 \delta _3 k^2}{2 \delta _1}\right) -\left( \frac{3 \alpha ^2 \delta _3 k^2}{2 \delta _1}\right) \left( (\tan (\xi )+\sec (\xi ))^2\right) \end{aligned}$$

**FAMILY-II**

91 $$\begin{aligned} a_0=-\frac{\alpha ^2 \delta _3 k^2}{2 \delta _1},~a_2=0,~a_1=0,~b_1=0,~b_2=-\frac{3 \alpha ^2 \delta _3 k^2}{2 \delta _1},~\omega =\frac{\alpha ^2 \delta _3+\beta ^2 \delta _4+\gamma ^2 \delta _5+\alpha ^4 \delta _3 k^2}{\alpha } \end{aligned}$$

**Figure 6:**
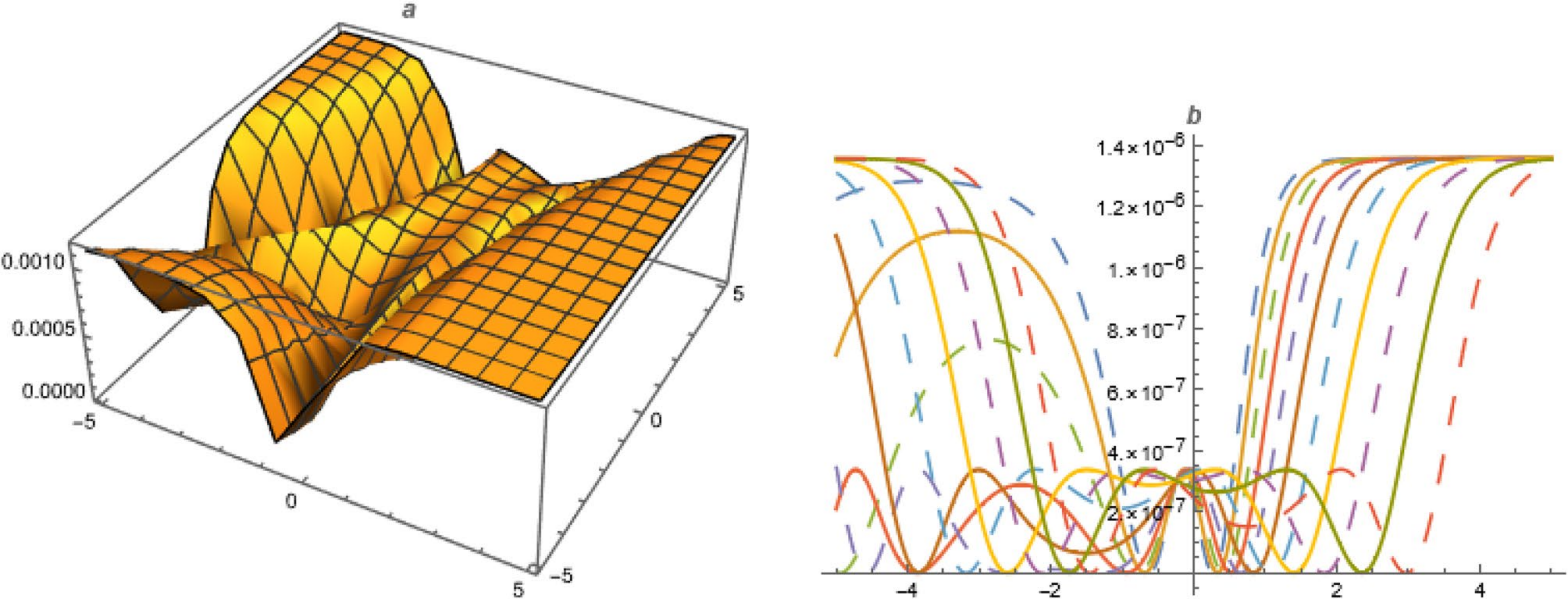
Solution $$U_{22}$$ with $$\alpha =0.1,~\beta =0.3,~\gamma =0.001,~\delta _1=1.01,~\delta _2=-1.3,~\delta _3=0.007,~\delta _4=0.1,~\delta _5=0.5,~k=4.1,~\mu =y=z=1,$$.

Put (91) in (72),

92 $$\begin{aligned} U_{26,2}=-\left( \frac{\alpha ^2 \delta _3 k^2}{2 \delta _1}\right) -\left( \frac{3 \alpha ^2 \delta _3 k^2}{2 \delta _1}\right) \left( \frac{1}{(\tan (\xi )+\sec (\xi ))^2}\right) \end{aligned}$$

**FAMILY-III**

93 $$\begin{aligned} a_0=\frac{\alpha ^2 \delta _3 k^2}{\delta _1},~a_2=-\frac{3 \alpha ^2 \delta _3 k^2}{2 \delta _1},~a_1=0,~b_1=0,~b_2=-\frac{3 \alpha ^2 \delta _3 k^2}{2 \delta _1},~\omega =\frac{\alpha ^2 \delta _3+\beta ^2 \delta _4+\gamma ^2 \delta _5+4 \alpha ^4 \delta _3 k^2}{\alpha } \end{aligned}$$

Put (93) in (72),

94 $$\begin{aligned} U_{26,3}=\left( \frac{\alpha ^2 \delta _3 k^2}{\delta _1}\right) -\left( \frac{3 \alpha ^2 \delta _3 k^2}{2 \delta _1}\right) (\tan (\xi )+\sec (\xi ))^2-\left( \frac{3 \alpha ^2 \delta _3 k^2}{2 \delta _1}\right) \left( \frac{1}{(\tan (\xi )+\sec (\xi ))^2}\right) \end{aligned}$$

**For A=C=-1/2, B=0, **

**FAMILY-I**

95 $$\begin{aligned} a_0=-\frac{3 \alpha ^2 \delta _3 k^2}{2 \delta _1},~a_2=-\frac{3 \alpha ^2 \delta _3 k^2}{2 \delta _1},~a_1=b_1=b_2=0,~\omega =\frac{\alpha ^2 \delta _3+\beta ^2 \delta _4+\gamma ^2 \delta _5+\alpha ^4 \delta _3 \left( -k^2\right) }{\alpha } \end{aligned}$$

Put (95) in (72),

96 $$\begin{aligned} U_{27,1}=-\left( \frac{3 \alpha ^2 \delta _3 k^2}{2 \delta _1}\right) -\left( \frac{3 \alpha ^2 \delta _3 k^2}{2 \delta _1}\right) \left( (\sec (\xi )-\tan (\xi ))^2\right) \end{aligned}$$

**FAMILY-II**

97 $$\begin{aligned} a_0=-\frac{\alpha ^2 \delta _3 k^2}{2 \delta _1},~a_2=0,~a_1=0,~b_1=0,~b_2=-\frac{3 \alpha ^2 \delta _3 k^2}{2 \delta _1},~\omega =\frac{\alpha ^2 \delta _3+\beta ^2 \delta _4+\gamma ^2 \delta _5+\alpha ^4 \delta _3 k^2}{\alpha } \end{aligned}$$

Put (97) in (72),

98 $$\begin{aligned} U_{27,2}=-\left( \frac{\alpha ^2 \delta _3 k^2}{2 \delta _1}\right) -\left( \frac{3 \alpha ^2 \delta _3 k^2}{2 \delta _1}\right) \left( \frac{1}{(\sec (\xi )-\tan (\xi ))^2}\right) \end{aligned}$$

**FAMILY-III**

99 $$\begin{aligned} a_0=\frac{\alpha ^2 \delta _3 k^2}{\delta _1},~a_2=-\frac{3 \alpha ^2 \delta _3 k^2}{2 \delta _1},~a_1=0,~b_1=0,~b_2=-\frac{3 \alpha ^2 \delta _3 k^2}{2 \delta _1},~\omega =\frac{\alpha ^2 \delta _3+\beta ^2 \delta _4+\gamma ^2 \delta _5+4 \alpha ^4 \delta _3 k^2}{\alpha } \end{aligned}$$

Put (99) in (72),

100 $$\begin{aligned} U_{27,3}=-\left( \frac{3 \alpha ^2 \delta _3 k^2}{2 \delta _1}\right) \left( \frac{1}{(\sec (\xi )-\tan (\xi ))^2}\right) +\left( \frac{\alpha ^2 \delta _3 k^2}{\delta _1}\right) -\left( \frac{3 \alpha ^2 \delta _3 k^2}{2 \delta _1}\right) \left( (\sec (\xi )-\tan (\xi ))^2\right) \end{aligned}$$

** For A=C=-1 B=0, **

**FAMILY-I**

101 $$\begin{aligned} a_0=-\frac{2 \alpha ^2 \delta _3 k^2}{\delta _1},~a_2=-\frac{6 \alpha ^2 \delta _3 k^2}{\delta _1},~a_1=b_1=b_2=0,~\omega =\frac{\alpha ^2 \delta _3+\beta ^2 \delta _4+\gamma ^2 \delta _5+4 \alpha ^4 \delta _3 k^2}{\alpha } \end{aligned}$$

Put (101) in (72),

102 $$\begin{aligned} U_{28,1}=-\left( \frac{2 \alpha ^2 \delta _3 k^2}{\delta _1}\right) \text {-}-\left( \frac{6 \alpha ^2 \delta _3 k^2}{\delta _1}\right) \left( \tan ^2(\xi )\right) \end{aligned}$$

**FAMILY-II**

103 $$\begin{aligned} a_0=-\frac{2 \alpha ^2 \delta _3 k^2}{\delta _1},~a_2=0,~a_1=0,b_1=0,~b_2=-\frac{6 \alpha ^2 \delta _3 k^2}{\delta _1},~\omega =\frac{\alpha ^2 \delta _3+\beta ^2 \delta _4+\gamma ^2 \delta _5+4 \alpha ^4 \delta _3 k^2}{\alpha } \end{aligned}$$

Put (103) in (72),

104 $$\begin{aligned} U_{28,2}=-\left( \frac{6 \alpha ^2 \delta _3 k^2}{\delta _1}\right) -\left( \frac{2 \alpha ^2 \delta _3 k^2}{\delta _1}\right) \left( \frac{1}{\tan ^2(\xi )}\right) \end{aligned}$$

**FAMILY-III**

105 $$\begin{aligned} a_0=-\frac{12 \alpha ^2 \delta _3 k^2}{\delta _1},~a_2=-\frac{6 \alpha ^2 \delta _3 k^2}{\delta _1},~a_1=0,~b_1=0,~b_2=-\frac{6 \alpha ^2 \delta _3 k^2}{\delta _1},~\omega =\frac{\alpha ^2 \delta _3+\beta ^2 \delta _4+\gamma ^2 \delta _5-16 \alpha ^4 \delta _3 k^2}{\alpha } \end{aligned}$$

Put (105) in (72),

106 $$\begin{aligned} U_{28,3}=-\left( \frac{12 \alpha ^2 \delta _3 k^2}{\delta _1}\right) -\left( \frac{6 \alpha ^2 \delta _3 k^2}{\delta _1}\right) \left( \tan ^2(\xi )\right) -\left( \frac{6 \alpha ^2 \delta _3 k^2}{\delta _1}\left( \frac{1}{\tan ^2(\xi )}\right) \right) \end{aligned}$$

**For A=0, B=1**

107 $$\begin{aligned} a_1=-\frac{6 \alpha ^2 C \delta _3 k^2}{\delta _1},~b_1=0,~b_2=0,~\omega =\frac{\alpha ^2 \delta _3+\beta ^2 \delta _4+\gamma ^2 \delta _5+\alpha ^4 \delta _3 \left( -k^2\right) }{\alpha } \end{aligned}$$

Put (107) in (72),

108 $$\begin{aligned} U_{29}=-\left( \frac{\alpha ^2 \delta _3 k^2}{\delta _1}\right) -\left( \frac{6 \alpha ^2 C \delta _3 k^2}{\delta _1}(-\frac{1}{\text {C}\xi }+\eta \text {) }\right) -\left( \frac{6 \alpha ^2 C^2 \delta _3 k^2}{\delta _1}\right) \left( \frac{1}{\text {C}\xi }+\eta \right) ^2 \end{aligned}$$

**B=C=0**

109 $$\begin{aligned} a_0=a_2=a_1=b_1=0,~b_2=-\frac{6 \alpha ^2 A^2 \delta _3 k^2}{\delta _1},~\omega =\frac{\alpha ^2 \delta _3+\beta ^2 \delta _4+\gamma ^2 \delta _5}{\alpha } \end{aligned}$$

Put (109) in (72),

110 $$\begin{aligned} U_{30}=-\left( \frac{6 \alpha ^2 A^2 \delta _3 k^2}{\delta _1}\right) \left( \frac{1}{A^2}\right) \end{aligned}$$

**For C=0**

111 $$\begin{aligned}&a_0=-\frac{\alpha ^2 B^2 \delta _3 k^2}{\delta _1},~a_2=0,~a_1=0,~b_1=-\frac{6 \alpha ^2 A B \delta _3 k^2}{\delta _1},\nonumber \\ {}&b_2=-\frac{6 \alpha ^2 A^2 \delta _3 k^2}{\delta _1},~\omega =\frac{\alpha ^2 \delta _3+\beta ^2 \delta _4+\alpha ^4 \left( -B^2\right) \delta _3 k^2+\gamma ^2 \delta _5}{\alpha } \end{aligned}$$

Put (111) in (72),

112 $$\begin{aligned} U_{31}=-\left( \frac{\alpha ^2 B^2 \delta _3 k^2}{\delta _1}\right) -\left( \frac{6 \alpha ^2 A B \delta _3 k^2}{\delta _1}\right) \left( \frac{B}{\exp (B \xi )-A}\right) -\left( \frac{6 \alpha ^2 A^2 \delta _3 k^2}{\delta _1}\right) \left( \frac{B}{\exp (B \xi )-A}\right) ^2 \end{aligned}$$

**Figure 7:**
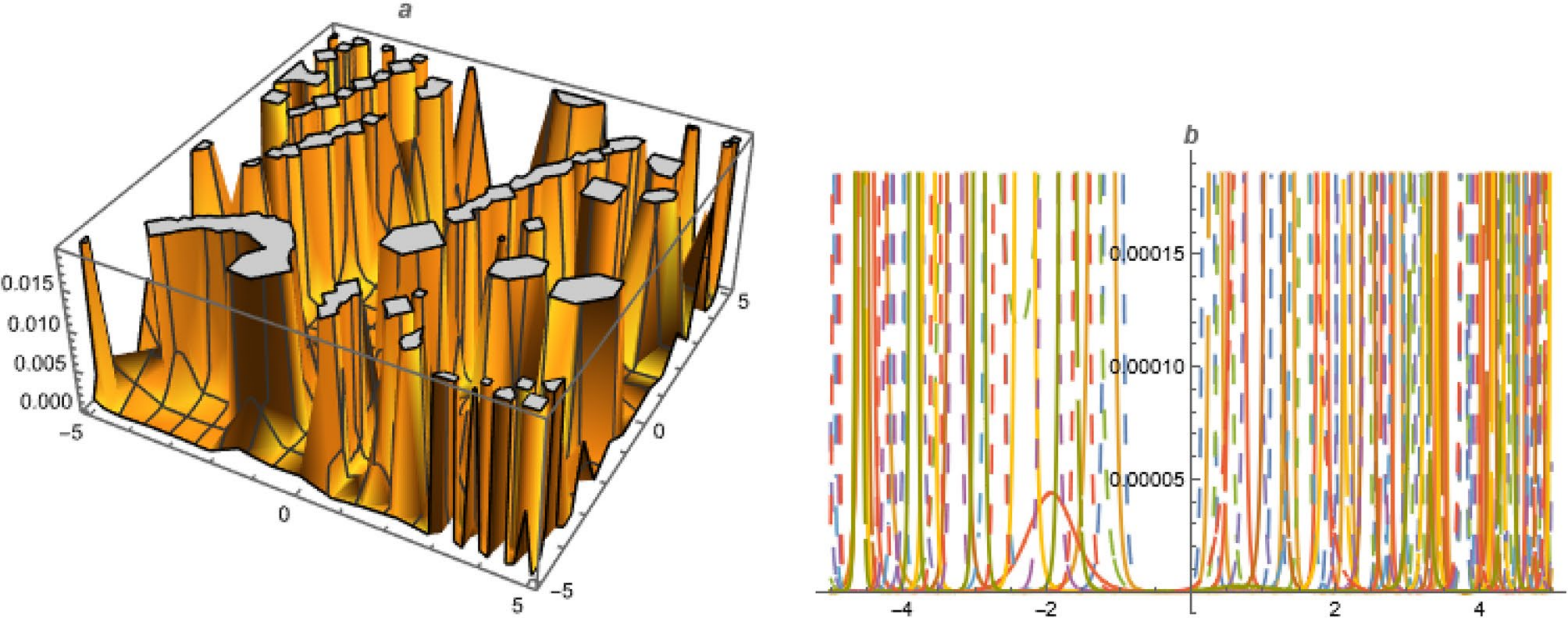
Solution $$U_{28,1}$$ with $$\alpha =0.2,~\beta =0.4,~\gamma =0.02,~\delta _1=1.03,~\delta _2=-1.4,~\delta _3=0.05,~\delta _4=0.2,~\delta _5=0.2,~k=2.1,~\mu =y=z=1.$$.

## Discussion of the results

In this section we have compared our investigated solutions with others solutions in different latest articles obtained solutions by using different techniques. Due to derive different values of $A{i},(i=-2,-1,0,1,2)$ in Eq. (38)and $A_{i}, B_{i}, C_{i}, D_{i} ,(i = 0,1,2)$ in Eq. (55) and $A_{N}, (N=1,2)$ in Eq. (60) and Eq. (65) respectively. Furthermore $a_{i},b_{i},(i = 1,2)$ in Eq. (72), we have achieved serval types results in the form of trigonometric, rational, exponential and rational functions. However, some of our constructed solutions are likely similar to due to the following pints.

$\bullet$ Our solutions ${U_1}$ and $U_{2}$ are likely similar to the solutions mentioned in Eqs. (16) and (18) respectively in^55^.

$\bullet$ Our solutions ${U_{14}}$ and $U_{15}$ are likely similar to the solutions mentioned in Eqs. (44) and (45) respectively in^56^.

$\bullet$ Our solutions ${U_{27,1}}$ and $U_{27,2}$ are likely similar to the solutions mentioned in Eqs. (73) and (81) respectively in^57^.

The residual overall constructed results are novel and have not been explored in any research literature. It is shown that our proposed methods provide an effective and a more powerful mathematical tool for solving nonlinear evolution equations in physical sciences. It is reliable and endorses a assortment of exact solutions NFPDEs.

## Conclusion

We have built abundant various exact solutions of Eq. (1) by employing the five mathematical approaches. All calculations and figures are handling by using the Mathematica 12.1 software. We conspired both 2-dimensional and 3-dimensional plots to understand physical behaviour of concern model by assigned certain value to the parameters in Figs. 1, 2, 3, 4, 5, 6 and 7. The offered mathematical techniques are more powerful and investigated results have many application in nonlinear science. The obtained results are in the form of trignometric, hypberbolic, exponential and rational forms. Engineers and researchers need to carefully evaluate the properties of the materials used and refine them to optimize the performance and efficiency of traveling wave solutions in various applications. Further research and development in this field can lead to improvements in hydrodynamics and fluids. We intend to use a variety of techniques in the future to study these nonlinear fractional wave equations in mathematical physics and find the lump, soliton, and breather solutions.

## Data Availability

The datasets used and/or analysed during the current study available from the corresponding author on reasonable request.
